# Experimental infection of duck origin virulent Newcastle disease virus strain in ducks

**DOI:** 10.1186/1746-6148-10-164

**Published:** 2014-07-17

**Authors:** Yabin Dai, Xu Cheng, Mei Liu, Xinyue Shen, Jianmei Li, Shengqing Yu, Jianmin Zou, Chan Ding

**Affiliations:** 1 Poultry Institute, Chinese Academy of Agricultural Sciences, 58 Cangjie Road, Yangzhou, Jiangsu 225125, China; 2 Jiangsu Co-innovation Center for Prevention and Control of Important Animal Infectious Diseases and Zoonosess, 88 South University Ave., Yangzhou, Jiangsu 225009, China; 3 Shanghai Veterinary Research Institute, Chinese Academy of Agricultural Sciences, 518 Ziyue Road, Shanghai 200241, China

**Keywords:** Newcastle disease virus, Duck, Breed, Age, Susceptibility, Pathogenesis, Virus shedding

## Abstract

**Background:**

Newcastle disease (ND) caused by virulent Newcastle disease virus (NDV) is an acute, highly contagious and fatal viral disease affecting most species of birds. Ducks are generally considered to be natural reservoirs or carriers of NDV while being resistant to NDV strains, even those most virulent for chickens; however, natural ND cases in ducks have been gradually increasing in recent years. In the present study, ducks of different breeds and ages were experimentally infected with duck origin virulent NDV strain duck/Jiangsu/JSD0812/2008 (JSD0812) by various routes to investigate the pathogenicity of NDV in ducks.

**Results:**

Six breeds (mallard, Gaoyou, Shaoxing, Jinding, Shanma, and Pekin ducks) were infected intramuscularly (IM) with JSD0812 strain at the dose of 5 × 10^8^ ELD_50_. Susceptibility to NDV infection among breeds varied, per morbidity and mortality. Mallard ducks were the most susceptible, and Pekin ducks the most resistant. Fifteen-, 30-, 45-, 60-, and 110-day-old Gaoyou ducks were infected with JSD0812 strain at the dose of 5 × 10^8^ ELD_50_ either IM or intranasally (IN) and intraocularly (IO), and their disease development, viral shedding, and virus tissue distribution were determined. The susceptibility of ducks to NDV infection decreased with age. Most deaths occurred in 15- and 30-day-old ducklings infected IM. Ducks infected IN and IO sometimes exhibited clinical signs, but seldom died. Clinical signs were primarily neurologic. Infected ducks could excrete infectious virus from the pharynx and/or cloaca for a short period, which varied with bird age or inoculation route; the longest period was about 7 days. The rate of virus isolation in tissues from infected ducks was generally low, even in those from dead birds, and it appeared to be unrelated to bird age and infection route.

**Conclusions:**

The results confirmed that some of the naturally occurring NDV virulent strains can cause the disease in ducks, and that ducks play an important role in the epidemiology of ND. The prevention of NDV spread in ducks should receive more attention and research in terms of preventing the occurrence and prevalence of ND.

## Background

Newcastle disease (ND) caused by virulent Newcastle disease virus (NDV) isolates is an acute, highly contagious, and fatal viral disease affecting most species of birds [1]. It is one of the most important avian viral diseases because of its economic impact on the poultry industry. The World Organisation for Animal Health (OIE) classifies it as a notifiable disease. First reported in 1926 [2,3], the disease still remains a worldwide epidemic and an important limiting factor in the development of commercial poultry production and the establishment of trade links [1].

NDV is capable of infecting a wide variety of avian species, and at least 241 species from 27 of the 50 orders of birds have been found to be susceptible to natural or experimental infections with NDV [4]. The virulence of NDV strains varies greatly with the host. Among poultry, chickens and turkeys are the most susceptible, and ducks and geese are the least susceptible. Ducks and geese are generally considered to be natural reservoirs or carriers of NDV while being resistant to NDV strains, even those most virulent for chickens [1]. However, outbreaks of ND have frequently occurred in geese throughout China since 1997, causing devastating economic losses [5-9]. Many NDV strains of different virulences have been isolated from diseased and clinically healthy ducks [10-25]. Some isolates were pathogenic for ducks, and natural ND cases in ducks have been gradually increasing in recent years [10,11,14,16-19,21,23-25], indicating that ducks may be not just reservoirs and carriers of NDV but also susceptible to infection with the disease.

During the winter of 2008, there were several outbreaks of ND in egg-laying duck farms in Jiangsu Province, and a virulent NDV strain duck/Jiangsu/JSD0812/2008 (JSD0812) highly pathogenic for chickens, geese, and ducks was isolated and identified [23,26,27]. In the present study, we investigate this strain’s pathogenicity in ducks through experimental infections of ducks of different breeds and ages. The role of ducks in the epidemiology of ND also was further explored by the detection of virus shedding. The results contribute toward a theoretical basis for comprehensive prevention and control of ND.

## Results

### Breed susceptibility

Six duck breeds were used to evaluate breed susceptibility to NDV JSD0812 strain in the present study. Under conditions of identical challenge doses, we found certain differences in morbidity and mortality among breeds (Table 1). Based on both morbidity and mortality, the mallard duck appeared to be the most susceptible, whereas the Pekin duck was the most resistant to NDV infection among the six breeds. All mallard ducklings showed clinical manifestations on day 2 postinoculation (PI) and died during the period of day 3 to day 5 PI. The mock-infected control birds remained healthy during the observation period.

**Table 1:** The pathogenicity of NDV JSD0812 strain in different duck breeds

**Breeds**	**Age (days)**	**Inoculation route**^**1**^	**Inoculation dose**	**Morbidity (%) (no. diseased/no. inoculated)**	**Mortality (%) (no. died/no. inoculated)**
Mallard duck	15	IM	5 × 10^8^ELD_50_	100 (10/10)	100 (10/10)
Gaoyou duck	15	IM	5 × 10^8^ELD_50_	100 (10/10)	60 (6/10)
Shaoxing duck	15	IM	5 × 10^8^ELD_50_	100 (10/10)	50 (5/10)
Shanma duck	15	IM	5 × 10^8^ELD_50_	100 (10/10)	10 (1/10)
Jinding duck	15	IM	5 × 10^8^ELD_50_	100 (10/10)	30 (3/10)
Pekin duck	15	IM	5 × 10^8^ELD_50_	60 (6/10)	10 (1/10)
Gaoyou duck	15	IM	Sterilized saline solution	0 (0/10)	0 (0/10)

^1^IM: inoculated intramuscularly.

### Age susceptibility

Gaoyou ducks of different ages infected with NDV JSD0812 strain either intramuscularly (IM) or intranasally (IN) and intraocularly (IO) showed clinical symptoms, but mortality rates varied with the age and inoculation route (Table 2). Deaths usually occurred in ducks inoculated IM, and their mortality had a tendency to decrease with increasing age. The mortalities of 15- and 30-day-old ducklings were 60% and 40%, respectively, and none or only a few of the 45-, 60- and 110-day-old birds died. However, in all ducks inoculated IN and IO, only one 110-day-old duck died on day 6 PI. This indicated that intramuscular inoculation of NDV was more likely to cause severe disease than intranasal and intraocular inoculation.

**Table 2:** The pathogenicity of NDV JSD0812 strain in Gaoyou ducks of different ages

**Age (days)**	**Inoculation route**^**1**^	**Inoculation dose**	**Morbidity (%) (no. diseased/no. inoculated)**	**Mortality (%) (no. died/no. inoculated)**	**Mean serum HI titer**^**2**^
15	IM	5 × 10^8^ ELD_50_	100 (10/10)	60 (6/10)	5.40 ± 0.84
	IN & IO	5 × 10^8^ ELD_50_	100 (10/10)	0 (0/10)	5.25 ± 0.96
30	IM	5 × 10^8^ ELD_50_	100 (10/10)	40 (4/10)	7.17 ± 0.75
	IN & IO	5 × 10^8^ ELD_50_	100 (10/10)	0 (0/10)	5.30 ± 0.95
45	IM	5 × 10^8^ ELD_50_	100 (10/10)	10 (1/10)	6.22 ± 0.97
	IN & IO	5 × 10^8^ ELD_50_	50 (5/10)	0 (0/10)	6.11 ± 1.05
60	IM	5 × 10^8^ ELD_50_	100 (10/10)	10 (1/10)	5.90 ± 0.74
	IN & IO	5 × 10^8^ ELD_50_	30 (3/10)	0 (0/10)	5.22 ± 1.20
110	IM	5 × 10^8^ ELD_50_	100 (10/10)	0 (0/10)	7.00 ± 1.22
	IN & IO	5 × 10^8^ ELD_50_	40 (4/10)	10 (1/10)	5.40 ± 1.26

^1^IM: inoculated intramuscularly; IN & IO: inoculated intranasally and intraocularly.

^2^Antibody detection was performed on day 15 PI and JSD0812 strain was used as antigen in HI tests. Values represent the mean ± SD and were expressed as log_2_ exponents.

Seroconversions were detected in all surviving ducks on day 15 PI in experiment 2. The antibody levels of ducks inoculated IM were usually somewhat higher than those of birds inoculated IN and IO (Table 2).

### Viral shedding

Viral shedding could be detected from some but not all infected ducks in experiment 2, and the duration and rate of viral shedding varied with inoculation route and age (Table 3). Generally, the durations and rates of viral shedding from ducks inoculated IN and IO were longer and higher than those from birds inoculated IM. With ducks inoculated IM, virus was detected in oropharyngeal and/or cloacal swabs from 15- and 30-day-old birds during the first 7 days and 3 days PI, respectively, and only in oropharyngeal swabs from 45-, 60-, and 110-day-old birds on day 2 PI. With ducks inoculated IN and IO, however, virus was detected in oropharyngeal and/or cloacal swabs during the first 7 days PI except 60-day-old birds in which virus was detected during the first 5 days PI. In addition, the duration and rate of viral shedding appeared to be related to bird age and tended to decrease with increasing age. This was pronounced in 45-, 60-, and 110-day-old ducks inoculated IM in which oropharyngeal shedding was limited to only 2 of 10 birds in each group.

**Table 3:** Virus isolations from swab samples of Gaoyou ducks of different ages inoculated either intramuscularly or intranasally and intraocularly

**Age (days)**	**Inoculation route**^**1**^	**Swab sample**	**Days PI**					
			**1**	**2**	**3**	**5**	**7**	**9 ~ 15**
15	IM	Oropharyngeal	+^2^ (7/10)^3^	+ (10/10)	+ (10/10)	+ (1/8)	+ (1/5)	-^4^ (0/4)
		Cloacal	– (0/10)	+ (2/10)	+ (8/10)	+ (3/8)	+ (2/5)	– (0/4)
	IN & IO	Oropharyngeal	+ (9/10)	+ (7/10)	+ (7/10)	– (0/10)	– (0/10)	– (0/10)
		Cloacal	+ (2/10)	+ (3/10)	+ (2/10)	+ (1/10)	+ (2/10)	– (0/10)
30	IM	Oropharyngeal	– (0/10)	+ (4/10)	– (3/10)	– (0/10)	– (0/7)	– (0/6)
		Cloacal	+ (1/10)	+ (1/10)	+ (2/10)	– (0/10)	– (0/7)	– (0/6)
	IN & IO	Oropharyngeal	+ (6/10)	+ (7/10)	+ (5/10)	– (0/10)	– (0/10)	– (0/10)
		Cloacal	+ (1/10)	+ (1/10)	+ (1/10)	+ (2/10)	+ (1/10)	– (0/10)
45	IM	Oropharyngeal	– (0/10)	+ (2/10)	– (0/10)	– (0/10)	– (0/9)	– (0/9)
		Cloacal	– (0/10)	– (0/10)	– (0/10)	– (0/10)	– (0/9)	– (0/9)
	IN & IO	Oropharyngeal	+ (8/10)	+ (4/10)	+ (4/10)	+ (1/10)	+ (1/10)	– (0/10)
		Cloacal	– (0/10)	– (0/10)	+ (2/10)	– (0/10)	+ (1/10)	– (0/10)
60	IM	Oropharyngeal	– (0/10)	+ (2/10)	– (0/10)	– (0/10)	– (0/9)	– (0/9)
		Cloacal	– (0/10)	– (0/10)	– (0/10)	– (0/10)	– (0/9)	– (0/9)
	IN & IO	Oropharyngeal	+ (5/10)	+ (6/10)	+ (2/10)	+ (1/10)	– (0/10)	– (0/10)
		Cloacal	– (0/10)	– (0/10)	– (0/10)	+ (1/10)	– (0/10)	– (0/10)
110	IM	Oropharyngeal	– (0/10)	+ (2/10)	– (0/10)	– (0/10)	– (0/10)	– (0/10)
		Cloacal	– (0/10)	– (0/10)	– (0/10)	– (0/10)	– (0/10)	– (0/10)
	IN & IO	Oropharyngeal	+ (4/10)	+ (2/10)	+ (2/10)	+ (3/10)	+ (1/9)	– (0/9)
		Cloacal	+ (1/10)	+ (1/10)	– (0/10)	+ (1/10)	– (0/9)	– (0/9)

^1^IM: inoculated intramuscularly; IN & IO: inoculated intranasally and intraocularly.

^2^At least one swab was virus isolation positive.

^3^Number of positive swabs/total number of swabs.

^4^All swabs were virus isolation negative.

The highest shedding rate usually occurred on days 2 and 3 PI, whether ducks were infected IM or IN and IO. In many cases, virus could not be detected concurrently in oropharyngeal and cloacal swabs from a bird on the same day. The virus isolation rate of oropharyngeal swab was generally higher than that of cloacal swab, indicating oropharynx may be a major excretion route of NDV.

### Virus tissue distribution

Virus detection in 12 tissue samples of ducks infected either IM or IN and IO on days 3 and 5 PI in experiment 3 are shown in Table 4. Overall, the virus isolation rates were relatively low and appeared to be unrelated to bird age and inoculation route, though they were higher in 15- and 45-day-old birds inoculated IM on day 3 PI. Meanwhile, there were also very rare cases in which virus could be detected synchronously in same tissue from both birds on the same day PI. In a total of 40 infected ducks, the rate of virus isolations was highest from bursa of Fabricius (30%); next from Harderian gland (25%), pancreas (20%), thymus (20%), and laryngotrachea (17.5%); and least from kidney (10%), spleen (5%), heart (5%), brain (2.5%), lung (2.5%), and small intestine (2.5%). No virus isolation was successful in liver samples.

**Table 4:** Virus distribution in tissues of Gaoyou ducks of different ages inoculated either intramuscularly or intranasally and intraocularly on days 3 and 5 PI

**Age (days)**	**Inoculation route**^**1**^	**Days PI**	**Brain**	**Live**	**Spleen**	**Pancreas**	**Heart**	**Kidney**	**Thymus**	**Harderian gland**	**Bursa of Fabricius**	**Lung**	**Laryngotrachea**	**Small intestine**
15	IM	3	-^2^ (0/2)^3^	– (0/2)	– (0/2)	– (0/2)	– (0/2)	+^4^ (1/2)	+ (2/2)	+ (2/2)	+ (2/2)	– (0/2)	+ (2/2)	+ (1/2)
		5	– (0/2)	– (0/2)	– (0/2)	– (0/2)	– (0/2)	– (0/2)	+ (1/2)	+ (2/2)	+ (2/2)	– (0/2)	+ (2/2)	– (0/2)
	IN & IO	3	– (0/2)	– (0/2)	– (0/2)	– (0/2)	– (0/2)	– (0/2)	– (0/2)	+ (1/2)	+ (1/2)	– (0/2)	+ (1/2)	– (0/2)
		5	– (0/2)	– (0/2)	– (0/2)	– (0/2)	– (0/2)	– (0/2)	+ (2/2)	– (0/2)	+ (2/2)	– (0/2)	– (0/2)	– (0/2)
30	IM	3	– (0/2)	– (0/2)	– (0/2)	+ (1/2)	– (0/2)	– (0/2)	– (0/2)	– (0/2)	+ (2/2)	– (0/2)	– (0/2)	– (0/2)
		5	– (0/2)	– (0/2)	– (0/2)	– (0/2)	– (0/2)	– (0/2)	– (0/2)	– (0/2)	– (0/2)	– (0/2)	– (0/2)	– (0/2)
	IN & IO	3	– (0/2)	– (0/2)	– (0/2)	+ (1/2)	+ (1/2)	+ (1/2)	– (0/2)	– (0/2)	+ (1/2)	– (0/2)	– (0/2)	– (0/2)
		5	– (0/2)	– (0/2)	– (0/2)	– (0/2)	– (0/2)	– (0/2)	– (0/2)	– (0/2)	– (0/2)	– (0/2)	– (0/2)	– (0/2)
45	IM	3	+ (1/2)	– (0/2)	+ (1/2)	– (0/2)	+ (1/2)	+ (1/2)	+ (2/2)	+ (1/2)	+ (1/2)	+ (1/2)	+ (1/2)	– (0/2)
		5	– (0/2)	– (0/2)	– (0/2)	+ (1/2)	– (0/2)	– (0/2)	– (0/2)	+ (1/2)	– (0/2)	– (0/2)	– (0/2)	– (0/2)
	IN & IO	3	– (0/2)	– (0/2)	+ (1/2)	– (0/2)	– (0/2)	– (0/2)	– (0/2)	– (0/2)	– (0/2)	– (0/2)	– (0/2)	– (0/2)
		5	– (0/2)	– (0/2)	– (0/2)	+ (1/2)	– (0/2)	– (0/2)	– (0/2)	+ (1/2)	– (0/2)	– (0/2)	– (0/2)	– (0/2)
60	IM	3	– (0/2)	– (0/2)	– (0/2)	– (0/2)	– (0/2)	– (0/2)	– (0/2)	+ (1/2)	– (0/2)	– (0/2)	+ (1/2)	– (0/2)
		5	– (0/2)	– (0/2)	– (0/2)	– (0/2)	– (0/2)	– (0/2)	– (0/2)	– (0/2)	– (0/2)	– (0/2)	– (0/2)	– (0/2)
	IN & IO	3	– (0/2)	– (0/2)	– (0/2)	+ (2/2)	– (0/2)	– (0/2)	– (0/2)	– (0/2)	– (0/2)	– (0/2)	– (0/2)	– (0/2)
		5	– (0/2)	– (0/2)	– (0/2)	+ (1/2)	– (0/2)	– (0/2)	– (0/2)	– (0/2)	– (0/2)	– (0/2)	– (0/2)	– (0/2)
110	IM	3	– (0/2)	– (0/2)	– (0/2)	– (0/2)	– (0/2)	+(1/2)	– (0/2)	– (0/2)	+(1/2)	– (0/2)	– (0/2)	– (0/2)
		5	– (0/2)	– (0/2)	– (0/2)	– (0/2)	– (0/2)	– (0/2)	– (0/2)	– (0/2)	– (0/2)	– (0/2)	– (0/2)	– (0/2)
	IN & IO	3	– (0/2)	– (0/2)	– (0/2)	+(1/2)	– (0/2)	– (0/2)	+ (1/2)	+ (1/2)	– (0/2)	– (0/2)	– (0/2)	– (0/2)
		5	– (0/2)	– (0/2)	– (0/2)	– (0/2)	– (0/2)	– (0/2)	– (0/2)	– (0/2)	– (0/2)	– (0/2)	– (0/2)	– (0/2)

^1^IM: inoculated intramuscularly; IN & IO: inoculated intranasally and intraocularly.

^2^Both samples were virus isolation negative.

^3^Number of positive samples/total number of samples.

^4^At least one sample was virus isolation positive.

Similarly, virus was seldom isolated from the tissue samples of dead birds in experiments 1 and 2, except for a 110-day-old Gaoyou duck inoculated IN and IO and died on day 6 PI in which virus could be isolated from laryngotrachea, pancreas, Harderian gland, bursa of Fabricius, and small intestine samples. The HA activity of all isolated viruses could be inhibited by NDV antiserum.

### Clinical signs

**Figure 1:**
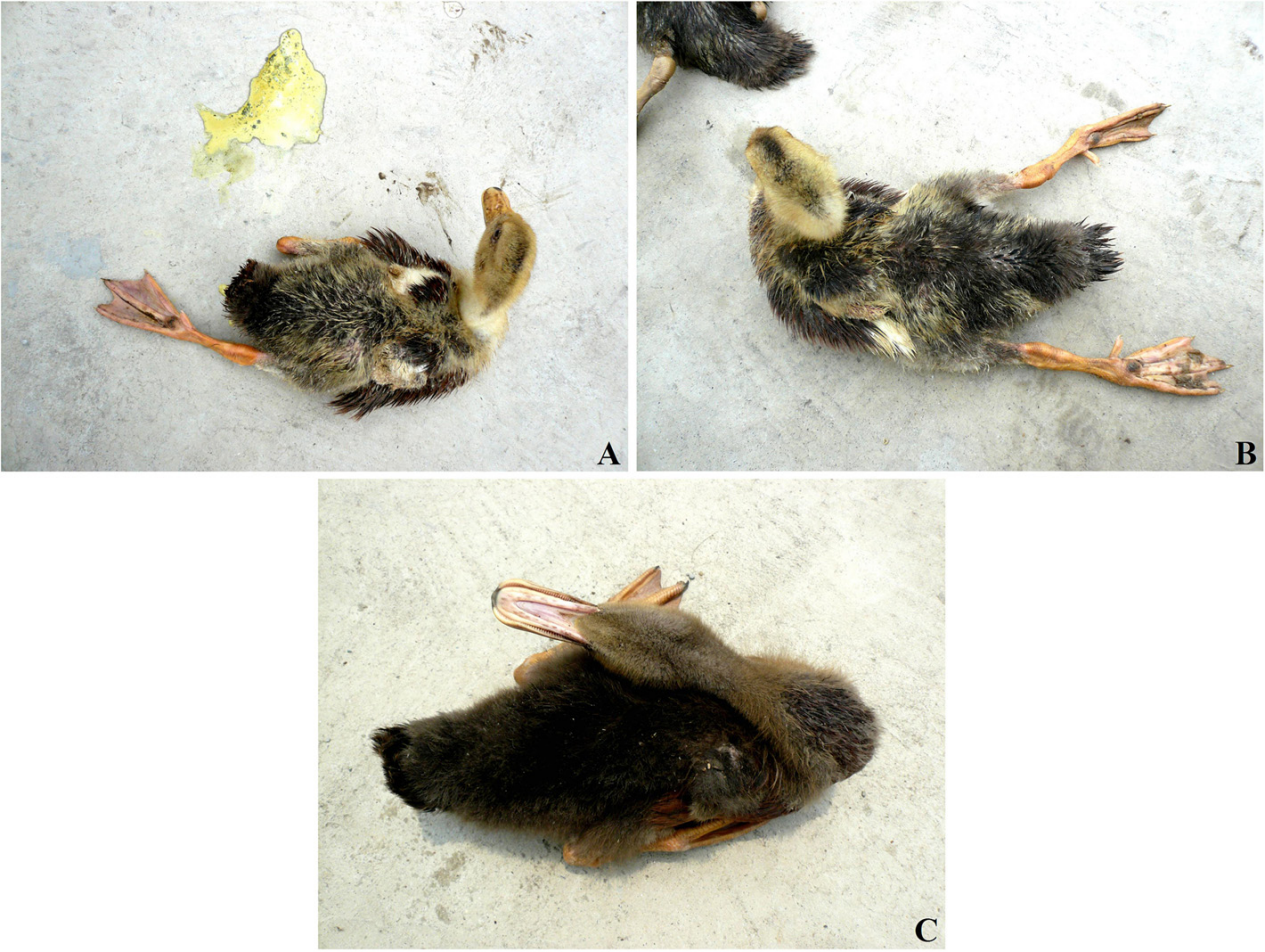
**Clinical signs of infected ducks. A**. Greenish-white diarrhea and unilateral leg paresis or paralysis in 15-day-old Shaoxing duck on day 3 PI. **B**. Bilateral leg paresis or paralysis in 15-day-old Shaoxing duck on day 4 PI. **C**. Twisting of head and neck in 15-day-old Gaoyou duck on day 4 PI.

Infected ducks usually began showing apparent symptoms on day 3 PI, though their elevated body temperature, excessively excreted oral mucus, and dried cloaca were observed as early as 1 day (24 h) PI when they were swabbed. Initially, ducks would show listlessness, anorexia, and watery, greenish-white diarrhea with foul odor (Figure 1A). Some birds began to show paresis or incomplete paralysis, including unilateral (Figure 1A) or bilateral (Figure 1B) weakness of the legs and wings on day 3 PI. They lay on their sternums with legs slightly extended to the sides, but could rise and stumblingly move away when approached. As the disease progressed, more severely affected birds were emaciated, unable to rise, and they lay on their sides and exhibited a swimming motion with both legs in vain attempts to escape when disturbed. By day 4 PI, some birds began to show other neurologic signs, including twisting of head and neck (Figure 1C), lack of muscular coordination, circling, and muscular tremors. Those birds displayed a possibly subconscious urge to eat or drink when they were near the feed or water troughs, but nothing was really ingested. Most deaths occurred during the period of day 4 to day 6 PI. The spirit and appetite of surviving birds were gradually restored to normal by day 8 PI in those which were only slightly affected, and by day 12 PI in those severely affected. The neurologic symptoms in the severely affected birds, if any, might have eased accordingly in the meantime, but the birds grew poorly. Moreover, in a few birds that showed paralysis during illness, one or both legs became maldeveloped or atrophied.

### Gross lesions

**Figure 2:**
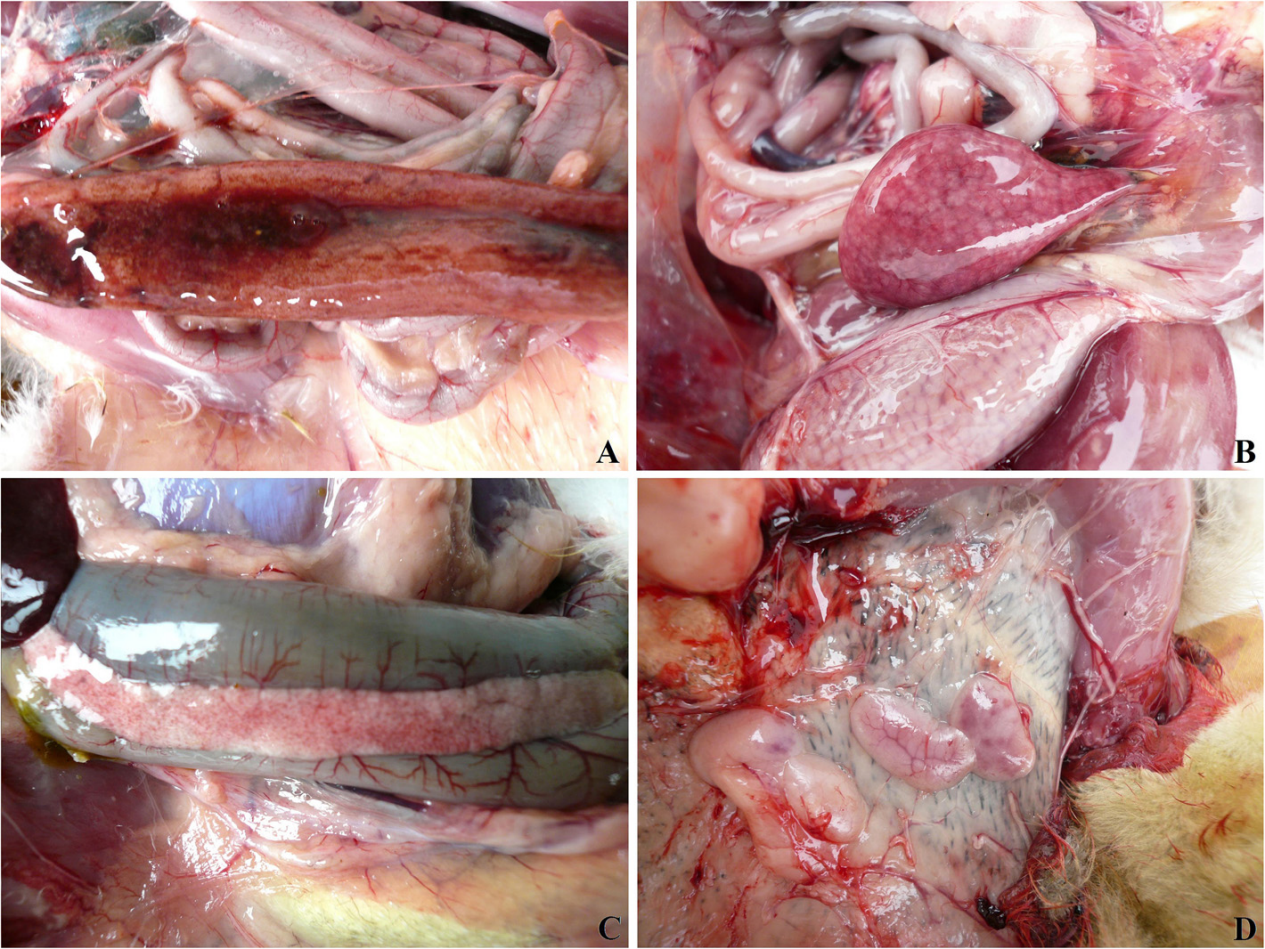
**Gross lesions of infected ducks. A**. Extensive hemorrhages in the mucosa of small intestine from 15-day-old mallard duck on day 5 PI. **B**. Hemorrhagic and mottled spleen from 15-day-old Gaoyou duck on day 9 PI. **C**. Congestion, hemorrhage, and necrosis in the pancreas from 15-day-old mallard duck on day 4 PI. **D**. Hemorrhagic thymuses from 15-day-old mallard duck on day 3 PI.

At necropsy, most dead birds showed no obvious gross lesions. Occasionally, mainly in some mallard ducklings, congestion or hemorrhages on the meninx and in the brain and diffuse brain edema were found. The caudal pharynx and tracheal mucosa were congested or hemorrhagic. Extensive hemorrhages were present in the mucosa of small intestine, with the most serious in the duodenum and upper part of the jejunum; intestinal contents were dark brown (Figure 2A). The spleens were a little atrophic or enlarged, friable, and hemorrhagic or mottled (Figure 2B). Congestion, hemorrhage, and necrosis could be found in the pancreas (Figure 2C). Severe hemorrhages or atrophy of thymus (Figure 2D) and bursa also occurred. Hemorrhages at the tip of the glands or petechiae in the mucosa of the proventriculus were infrequently found.

## Discussion

In this study, the morbidity and mortality of 15-day-old mallard, Gaoyou, Shaoxing, Shanma, Jinding, and Pekin ducklings challenged with JSD0812 strain differed, indicating that susceptibility to this strain varies between breeds. Shi, *et al*. also found the mortality of 10-day-old white-feather Muscovy, black-feather Muscovy, Pekin, and mule ducklings was 66.7%, 40%, 46.7%, and 6.67%, respectively, after they infected IM with a Muscovy duck origin velogenic NDV strain FP1/02 at the dose of 2.5 × 10^9.5^ ELD_50_ per bird [24]. Those results confirmed the existence of certain differences in the susceptibility to NDV among duck breeds. Our data also indicate that the susceptibility of ducks to NDV is related to age and inoculation route. The susceptibility of mallards to NDV has been reported to be reduced substantially with increasing age, and the mortalities of infected birds differed with inoculation routes [28]. In the present study, ducklings less than 30 days old were more susceptible based on morbidity, mortality and viral shedding, and ducks could be killed only when they were inoculated IM.

The domestic ducks were reported to be moderately susceptible to NDV. Lentogenic and mesogenic vaccine strains replicated only in the pharynx and were excreted from the pharynx (the infection site) for short periods, and at low infective titers. The naturally occurring velogenic strains could cause viremia, and they were excreted for approximately a week [29]. In our study, ducks infected with JSD0812 strain either IM or IN and IO were also found to shed virus from the pharynx and/or the cloaca for a short period; the longest period was about 7 days. Antibody plays an important role in inhibition of virus replication. Birds infected IM usually produced an earlier and higher level of HI antibody than those infected IN and IO; this is perhaps a reason that the period and rate of viral shedding in ducks infected IM were shorter and lower than those in birds infected IN and IO.

A low rate of virus isolation in tissues was found in mallards that died from infection of NDV Texas GB strain, a velogenic virus commonly used as a standard challenge virus in efficacy and potency testing of ND vaccines, and in research in the United States. The rate of isolations was greatest from brain (30.3%), next from lung (10.4%), and least from liver-spleen suspension (0.8%) [28]. This study showed a similar result in the low rate of virus isolation in tissues from ducks infected with JSD0812 strain. Baffling to us was that the rate of virus isolation in tissues was relatively low in infected ducks, even though some of those birds exhibited clinical signs of disease or died. We speculated that NDV only underwent limited replication and persisted for a short period in tissues, or it replicated at very low level so as not to be detected by virus isolation in SPF chicken embryos, though Frend & Trainer suggested that mortality of ducks from NDV might be caused by “viral toxicity” rather than the result of a classical infectious process; the toxic action is due to a partial cycle of replication that leads to the death of the infected cell, but not to the production of infective progeny [28]. In recent years, we have encountered many cases in which dead ducks had exhibited obvious clinical signs and gross lesions, and our attempts to isolate virus from tissue samples were unsuccessful, making it difficult to make accurate diagnoses. These results may provide us with valuable clues to disease diagnoses.

The observations in the present study suggest that clinical manifestations of infected ducks were primarily neurologic, and essentially in agreement with those reported in mallards experimentally infected with Texas GB strain [28]. In this study, we noted that some ducks surviving the experimental periods showed poor subsequent growth or maldevelopment of one or both legs. It is concluded that within extensive production systems, most birds that survive the disease may die eventually through difficulty eating and drinking over a long period, through secondary infection by other pathogens, or by trampling by other birds; subsequently leading to an increase in mortality in flocks.

There is still debate over whether NDV can cause the disease in ducks [30,31], though many natural outbreaks were associated with NDV [10,11,14,16-19,21,23-25], and ducks had developed the disease or died after experimental infections with virulent strains at large doses [23,24,29]. Combining the results from this and other studies on ducks, some of the inconsistencies may be due to differences in NDV strain, dose and route of inoculation, breed, age, and maternal antibody level in different experiments. Especially, doses of the virus lethal to chickens or geese, and the natural routes of infection (inhalation, ingestion, conjunctiva, *etc*.), perhaps do not cause illness and death in ducks because ducks are more resistant to NDV infection compared to chickens and geese.

It is noteworthy that the JSD0812 strain used in this study was isolated from an outbreak in a laying-duck flock. During the outbreak, several duck farms in local area were affected. In the affected flocks, egg production sharply declined by about 70%, the morbidity was about 80%, and the mortality varied from 30% to 50%. The diseased birds showed diarrhea, and nervous signs, and the dead birds mainly manifested by focal hemorrhage and necrosis of the intestinal mucosa, and congestion and hemorrhage of the ovarian follicles [23]. In our previous experiments, this strain was highly virulent for chickens, geese, and ducks [23,24,27]. Some strains caused morbidity and mortality only when ducks were infected with high doses under experimental conditions [24,27,28], and though ducks may have little chance of exposure to such large amounts of virus at a time under natural conditions, the situation is not impossible, particularly in farms with low to minimal biosecurity.

## Conclusions

The results of the present study demonstrated that duck origin NDV JSD0812 strain could cause disease in ducks, and ducks play an important role in the epidemiology of ND. Considering the growing number of ND cases in ducks in recent years, some of the naturally occurring NDV virulent strains already have certain capability to produce disease in ducks, and, no matter where the viruses originated or how they mutated, the prevention of their spread in ducks should receive more attention and research in terms of preventing the occurrence and prevalence of ND.

## Methods

### Virus strain, serum and antigen

The NDV filed strain JSD0812 was isolated during an outbreak from a laying-duck flock, and identified as velogenic genotype VII strain. It had MDT, ICPI and IVPI of 54.6 h, 1.75, and 2.68, respectively [23]. The standard NDV positive antiserum was purchased from Harbin Weike Biotechnology Development Co., Ltd. (Harbin, China).

### Specific pathogen free (SPF) chicken embryos

SPF fertile chicken eggs were purchased from Beijing Merial Vital Laboratory Animal Technology Co., Ltd., Beijing, China (production license: SCXK (Jing) 2009 0003), and incubated for 9–10 days.

### Experimental design

One-day-old ducklings were obtained from farmers in Yangzhou, China. The parents had not been immunized with any ND vaccine, and their NDV antibodies tested negative. The ducklings were identified by wing bands and raised to the desired age in semi-isolation facilities. Before experimental infection, all ducks tested negative for maternal antibody against NDV. All animal experiments were approved by the Experimental Animal Administration Committee of Jiangsu Province, China, and performed in accordance with the guidelines for animal experiments of Jiangsu Province, China.

#### ***Experiment 1***

Six breeds, including mallard, Gaoyou, Shaoxing, Jinding, Shanma, and Pekin duck, were taken to investigate breed susceptibility to NDV. At 15 days of age, groups of 10 ducklings were each inoculated IM with 0.2 mL of allantoic fluid containing 5 × 10^8^ ELD_50_ virus. Ten Gaoyou ducklings were mock inoculated with sterilized saline solution as a control. The experiment period was 15 days.

#### ***Experiment 2***

To assess age susceptibility and virus shedding, 15-, 30-, 45-, 60- and 110-day-old Gaoyou ducks, the most popular egg-laying duck breed locally, in groups of 10 birds each were infected with NDV JSD0812 strain at a dose of 5 × 10^8^ ELD_50_ in a volume of 0.2 mL per bird, either IM or IN and IO. Oropharyngeal and cloacal swabs were taken from each bird daily for the first 3 days, and thereafter every 2 days until day 15 PI. Swabs were placed in Eppendorf tubes containing 0.5 mL PBS (0.01 mol/L, pH 7.4). At the end of the experiment period, blood samples were collected from all surviving birds for serological testing.

#### ***Experiment 3***

To determine virus distribution, 15-, 30-, 45-, 60- and 110-day-old Gaoyou ducks in groups of 4 birds each were infected with NDV JSD0812 strain at a dose of 5 × 10^8^ ELD_50_ in a volume of 0.2 mL per bird, either IM or IN and IO. Two ducks from each group were killed on days 3 and 5 PI. Tissue samples, including brain, heart, liver, spleen, pancreas, kidney, lung, laryngotrachea (including throat and trachea) and small intestine (including contents), Harderian glands, thymus, and bursa of Fabricius, were removed aseptically for virus re-isolation in SPF chicken embryos.

During the periods of the above experiments, each group was maintained in a cage in a separate disinfected room, and all groups received the same feeding and husbandry: they fed and drank *ad libitum*, and their waste was cleaned regularly. Except for ducks in experiment 3, the birds were inspected a minimum of three times daily for the development of clinical signs of the disease or for mortality. Ducks that manifested listlessness, inappetence, greenish or white diarrhea, and paresis or incomplete paralysis were considered to be morbid. Immediate postmortem examination of dead birds was performed, and tissue samples, such as intestine, liver, spleen, pancreas, laryngotrachea, *etc*., were collected aseptically for virus re-isolation. All swab, serum, and tissue samples were stored at -20°C until further use.

### Virus isolation

The tissue samples were ground completely and added to sterile saline at the ratio of 1:4 (V/V). The swab and three-times freeze-thawed tissue samples were centrifuged at 6700 × *g* for 5 min. Penicillin and streptomycin were added to the supernatants to final concentrations of 2000 IU/mL and 2 mg/mL, respectively, and inoculated into 9- to 10-day-old SPF chicken embryos via allantoic cavity route, using 2 embryos per sample (0.2 mL/embryo). The inoculated embryos were incubated for 120 h, and candled twice daily after 24 h PI. The allantoic fluids of embryos that died within 24–120 h and of those still alive at 120 h were harvested, and tested for haemagglutination (HA) activities. Fluids that resulted in a negative reaction were re-tested. If the third-generation fluid of a sample still tested HA negative, its virus isolation was determined to be negative.

### Haemagglutination (HA) and haemagglutination inhibition (HI) tests

HA activity of allantoic fluid and antibody titer against NDV in serum were determined by HA and HI tests, respectively, as recommended by OIE [32]. However, duck red blood cells were used as instruction cells, and NDV JSD0812 strain homologous to that used for inoculation was used as antigen in HI tests. Positive and negative control antigens and antisera were run with each HI test. The antibody titers were expressed in log2 units.

## Abbreviations

HA: Hemagglutination; HI: Hemagglutination inhibition; ELD_50_: 50% embryo lethal dose; ICPI: Intracerebral pathogenicity index in day-old SPF chickens; IM: Intramuscularly; IN: Intranasally; IO: Intraocularly; IVPI: Intravenous pathogenicity index in 6-week-old SPF chickens; ND: Newcastle disease; MDT: Mean death time (hours) for 10-day-old SPF chicken embryos; NDV: Newcastle disease virus; PI: Postinoculation; SPF: Specific-pathogen-free.

## Competing interests

The authors declare they have no competing interests.

## Authors’ contributions

Conceived and designed the experiments: YD, SY, CD, JZ. Performed the experiments: YD, XC, ML, XY, JL. Analyzed the data: YD, XC, ML. Wrote the paper: YD, CD. All authors read and approved the final manuscript.
